# Roles of Macrophage Subtypes in Bowel Anastomotic Healing and Anastomotic Leakage

**DOI:** 10.1155/2018/6827237

**Published:** 2018-02-18

**Authors:** Jinyao Shi, Zhouqiao Wu, Ziyu Li, Jiafu Ji

**Affiliations:** Gastrointestinal Cancer Center, Key Laboratory of Carcinogenesis and Translational Research (Ministry of Education), Peking University Cancer Hospital & Institute, No. 52 Fu-Cheng Road, Hai-Dian District, Beijing 100142, China

## Abstract

Macrophages play an important role in host defense, in addition to the powerful ability to phagocytose pathogens or foreign matters. They fulfill a variety of roles in immune regulation, wound healing, and tissue homeostasis preservation. Macrophages are characterized by high heterogeneity, which can polarize into at least two major extremes, M1-type macrophages (classical activation) which are normally derived from monocytes and M2-type macrophages (alternative activation) which are mostly those tissue-resident macrophages. Based on the wound healing process in skin, the previous studies have documented how these different subtypes of macrophages participate in tissue repair and remodeling, while the mechanism of macrophages in bowel anastomotic healing has not yet been established. This review summarizes the currently available evidence regarding the different roles of polarized macrophages in the physiological course of anastomotic healing and their pathological roles in anastomotic leakage, the most dangerous complication after gastrointestinal surgery.

## 1. Introduction

Macrophages are myeloid immune cells that play a central role in inflammation and host defense [1, 2]. These cells are characterized by the powerful ability of phagocytosis and are credited with protecting the host from infection through a process so-called “innate immunity” [3]. In recent years, with the accumulation of evidence, macrophages have emerged as one of the most versatile cells. Their roles have shifted from immune effector cells which conduct host defense just as “trashmen” to predominant “directors” and “executors” for regulating inflammatory response, keeping tissue homeostasis, participating in wound healing and tissue remodeling [4].

Macrophages are actively involved in the wound healing process, while their role in a special surgical wound, also known as the anastomotic wound, has not yet been fully established. Anastomosis is constructed after removal of gastrointestinal tumor or bowel resection by surgeons to reconstruct the continuation of the gastrointestinal tract. Abnormal healing of anastomosis may develop into anastomotic leakage (AL), defined as luminal contents leaking from a surgical bowel connection [5]. It is the most dangerous complication after colorectal surgery [6–8], because it is responsible for up to 40% postoperative mortality rate, prolonged hospitalization, and an increase in the cost of healthcare due to the treatment of sepsis and the need for reoperation [9].

From a macroscopic point of view, the cause of AL mainly includes communication, infection, and healing disturbances [10]. However, a detailed mechanism on a cellular level is yet to be established due to the limited evidence. In this review, basing on heterogeneous populations of macrophages and their opposed tendencies of polarization, we tend to discuss the roles of different types of macrophages in an uneventful anastomotic healing and their pathological roles in anastomotic leakage.

### 1.1. Subtypes of Macrophages

Macrophages or mononuclear phagocytes had been long thought to originate from hematopoietic stem cells (HSCs). The prevailing dogma has stated that all macrophages derived from and were also replenished by monocytes [11]. However, macrophage family cells (cells of the mononuclear phagocyte system) manifest remarkable heterogeneity, in both their morphology and biological functions [12, 13]. These recent data have challenged the long-held conception about “HSC-monocyte-macrophage.” Evidence showed that tissue-resident macrophages like Kupffer cells of the liver, epidermal Langerhans cells of the skin, and microglia of the brain derived from a yolk sac and could persist in adult mice independent of HSCs [14–21]. Those tissue-resident macrophages can renew in situ, although they might also be replenished by blood monocytes in certain situations. In contrast to monocyte-derived macrophages which participate in an antibacterial process during acute inflammatory response, tissue-resident macrophages express different functional properties and play a central role in maintaining tissue architecture, function, and homeostasis [22–25], and their role in anastomotic healing is further discussed below.

The diversity and plasticity were recognized as hallmarks of macrophages, which contribute to their significant heterogeneity. In general, polarization of macrophages can be divided into two major extremes, that is, the classical activation which results in M1-type macrophages (M1) and the alternative activation which results in M2-type macrophages (M2). Those two types of macrophages perform diverse functional phenotypes in response to microenvironmental signals, like microbial products, damaged cells, and cytokines from activated lymphocytes. Specifically, ligands of Toll-like receptors (TLRs) and interferon-*γ* (IFN-γ) can induce macrophages to polarize into M1-type macrophages; on the contrary, interleukin-4 (IL-4) and interleukin-13 (IL-13) induce macrophages to polarize into M2-type macrophages [26–28]. However, such explanation may not fully illustrate all different activation scenarios. Murray et al. proposed that there should be some other subtypes between M1 and M2 [29], including the M2a subgroup induced by IL-4 and IL-13, the M2b subgroup activated by immune complexes (TLRs), and the M2c subgroup deactivated by glucocorticoids, transforming growth factor (TGF), or interleukin-10 (IL-10) [30, 31]. Moreover, it is also reported that there might be a supplementary subtype of M2 (M2d), which is elicited by TLR agonists and adenosine [32, 33]. It seems that the polarization of macrophages should be viewed as a continued spectrum, on which, two types of macrophages (M1 and M2) occupied the opposite ends. Another classification of polarization proposed by Mosser and Edwards suggested that macrophages are activated to form three populations in charge of host defense, wound healing, and immune regulation, respectively [34]. The authors classified macrophages on the basis of their fundamental functions rather than of the stimuli. Matching with the previously discussed conception of “M1-M2” paradigm, most of monocyte-derived macrophages are classically activated and express the M1 phenotype, which exerts host defense; reversely, tissue-resident macrophages are mainly activated in the alternative pathway which expresses M2-like characteristic and preserves tissue homeostasis and resolution of inflammation [21–23].

### 1.2. The Role of Polarized Macrophages in Physiological Anastomotic Healing

The wall of the alimentary tract contains four layers (i.e., mucosa, submucosa, muscularis propria, and serosa). For a classic end-to-end inverted bowel anastomosis, apposition of the serosa vanishes the gap between the two ends of the gastrointestinal tract, providing a barrier that insulates the sterile abdominal cavity from luminal contents and bacteria; moreover, this layer is important in providing a matrix for fibroblasts [35]. The submucosa consists of blood vessels, lymphatics, and nerve fibers; this layer is the source of fibroblasts that become active after gastrointestinal surgery and start to deposit collagen. The stapled or sutured collagen fibers in this layer provide most of the tensile strength of anastomosis [36]; hence, the submucosa is of great importance in anastomotic healing. The mucosal layer also plays a role in maintaining homeostasis to allow the healing process. A pool of macrophages in the gastrointestinal mucosa is the largest pool of tissue macrophages in the body, and a long-lasting macrophage absence or dysfunction impairs anastomotic healing [37, 38].

**Figure 1:**
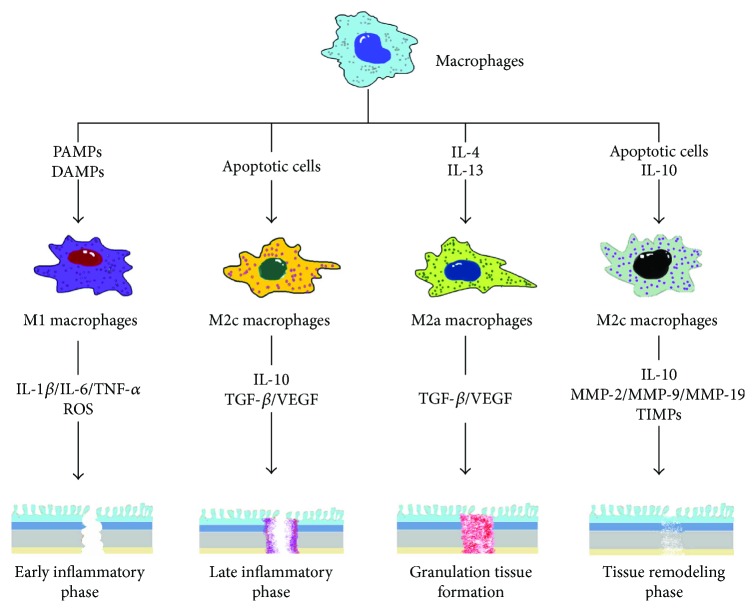
Polarization of macrophages in normal healing of anastomosis. Inactivated macrophages can be stimulated by various stimuli (e.g., PAMP, DAMP/IL-4, and IL-13/apoptotic cell) and polarize into M1- or M2- (M2a, M2c) type macrophages during different phases of normal anastomotic healing. Differentiated macrophages express a variety of cytokines (e.g., IL-1*β*, IL-6, IL-10, and TNF-*α*), growth factors (e.g., VEGF), and enzymes (MMPs). These biochemical substances acting upon tissues contribute to tissue repair and remodeling. PAMP: pathogen-associated modifying patterns; DAMP: damage-associated modifying patterns; IL: interleukin; TNF-*α*: tumor necrosis factor-*α*; VEGF: vascular endothelial growth factor; MMPs: matrix metalloproteinases.

Tissue repair and healing after injury have been studied for centuries but remain understood to a limited object, that is, skin. Different from that, healing of the gastrointestinal tract is anatomically obscured from inspection, only allowing the surgeon to judge the success of the operation only on the patient's parameters of general wellbeing [36]. There are some differences between the skin and anastomotic healing including anatomy (e.g., no equivalent anatomic component of the serosa in cutaneous tissues) and collagen and collagenase activity [39]. However, classical response to injury occurs in all organs and tissues. The physiological course of anastomotic healing can also be divided into three overlapping but distinct stages, which include inflammation, new tissue formation, and remodeling (Figure 1) [40, 41].

#### 1.2.1. Inflammation

In addition to infection of diverse microbial factors, injuries or traumas such as surgical strike can also lead to a non-pathogen-associated inflammation, which can be further divided into the early inflammatory response and the late one [42]. In the early inflammatory phase, neutrophils are recruited from circulating blood to local wounding tissue (the anastomotic area) at first. Those recruited polymorphonuclear cells remove the local foreign particles or bacteria and then undergo apoptosis or necrosis. After that, monocytes are recruited and differentiate into macrophages which are highly phagocytic. They phagocytose impaired neutrophils and other tissue debris to protect from further tissue damage. During this phase, in response to pathogen-associated modifying patterns (PAMPs) in a contaminative circumstance or damage-associated modifying patterns (DAMPs) in a sterile circumstance, macrophages are classically activated and express the M1 phenotype [43–45]. M1 macrophages release high concentrations of proinflammatory cytokines such as tumor necrosis factor-*α* (TNF-*α*), interleukin-1*β* (IL-1*β*), interleukin-6 (IL-6), and interleukin-12 (IL-12); protease; and reactive oxygen species (ROS) [34], all of which are thought to be important for microbial killing and proinflammatory response [13]. M1 macrophages can also produce collagenase, a high-activity enzyme that causes collagen degradation that results in low anastomotic strength early after the formation of an anastomosis [46]. In the late inflammation phase, with excessive phagocytosis of apoptotic neutrophils, engagement of *β*2 integrins on macrophages by apoptotic neutrophils activates macrophages to express anti-inflammatory mediator transforming growth factor (TGF) [47]. In contrast, the production of proinflammatory cytokines like TNF-*α* and IL-1*β* was inhibited [48, 49]. Thus, the phenotype of macrophages switches from proinflammatory M1-like to anti-inflammatory M2-like. These macrophages produce cytokines such as IL-10 and lay the foundation for new tissue formation by secreting other growth factors such as vascular endothelial growth factor (VEGF) [50, 51]. Because macrophages stimulated with IL-10, TGF, or glucocorticoids in vitro polarize into the M2c subtype that shares similarities with anti-inflammatory macrophages [30, 52–58], it suggests that anti-inflammatory macrophages belong to M2c-type macrophages and are able to amplify their anti-inflammatory response by secreting IL-10 and TGF in a feedforward loop. In addition, anti-inflammatory and regenerative capacities of anti-inflammatory macrophages were shown to be entirely IL-10-dependent in sterile environments, for example, in surgical wound [59].

#### 1.2.2. New Tissue Formation

In this phase, macrophages resident in tissue or recruited from peripheral blood, known as profibrotic macrophages, generate various growth factors such as TGF, platelet-derived growth factor (PDGF), fibroblast growth factor-2, or insulin-like growth factor-1 [60]. Among them, TGF is a profibrotic cytokine that exerts on fibroblasts and activates them to differentiate into myofibroblasts in wound tissue. Myofibroblasts produce a mass of extracellular matrix (ECM) components including collagen and fibronectin to fill up the tissue defect. For the gastrointestinal tract, collagen can also be produced by smooth muscle cells [61]. Collagen subtypes in the gastrointestinal tract are collagens I, III, and V, compared to solely collagen I and III in the skin [62]. By efficient contractile forces from myofibroblasts, fractured wound tissue can be bound together and rebuild their integrity [63]. Meanwhile, profibrotic macrophages and activated fibroblasts release proangiogenic factors like VEGF, which elicit endothelial progenitor cells crawling towards wound tissue, to promote new vessel formation (angiogenesis). Invasion of the capillary increases the blood supply to local tissues and facilitates anastomotic healing. Furthermore, studies of the healing colonic mucosa of rabbits after experimental excision showed that an abundance of mesenchymal cells in the healing intestinal muscle layers accompanies capillary invasion; these cells can differentiate into smooth muscle cells and histiocytes, which are thought to be responsible for the reestablishment of smooth muscle tissue [64, 65]. Profibrotic macrophages, myofibroblasts, and neovessels all together constitute granulation tissue, the most important fundamental compartment in the normal course of wound healing [40, 41]. These profibrotic macrophages are functionally classified as M2a-like macrophages because they can be induced in vitro by IL-4 and IL-13 [23, 30]. However, it is not clear whether anti-inflammatory and profibrotic macrophages can be clearly distinguished in vivo, and it appears that macrophage plasticity creates a mixture or continuous variant shifts during wound healing [50].

#### 1.2.3. Remodeling

Remodeling of anastomosis is a dynamic process of maturation within healed tissue that is based on a balance between ECM deposition and breakdown and tissue remodeling [66, 67]. A part of tissue-resident macrophages termed as fibrolytic macrophages is critical for maintaining this dynamic balance. They produce matrix metalloproteinases like matrix metalloproteinase-2 (MMP2), matrix metalloproteinase-9 (MMP9), matrix metalloproteinase-12 (MMP12), and matrix metalloproteinase-19 (MMP19) [42, 68], to degrade matrix macromolecules, that is, collagen, one of the most important components of ECM. The submucosa is a strength layer of the gastrointestinal tract and made predominantly of collagen, and remodeling of this layer predominates the strength of the anastomosis. Depending on MMPs secreted by fibrolytic macrophages, initially deposited collagen fibers are rearranged and cross-linked, remodeled from type III collagen to type I collagen; the latter one is much stronger. Besides, fibrolytic macrophages also regulate the degradation by synthesizing the tissue inhibitor of metalloproteinases (TIMPs), which can inhibit the activities of MMPs. Furthermore, fibrolytic macrophages are responsible for the induction of fibroblast apoptosis, subsequent removal of apoptotic cells, and suppression of further inflammation via IL-10 release [60]. Fibrolytic macrophages are proposed to be classified as M2c-like macrophages which can be elicited in vitro by apoptotic cells and IL-10 [69, 70].

Thus, macrophages participate in whole physiological courses of anastomotic healing. Among the three main phases of tissue repair, macrophages express different phenotypes during different stages. There are at least four kinds of macrophages in a condition of normal tissue repair: (1) proinflammatory macrophages, (2) anti-inflammatory macrophages, (3) profibrotic macrophages, and (4) fibrolytic macrophages. If we sort out those four kinds of macrophages according to “M1-M2” paradigm, proinflammatory and profibrotic macrophages may, respectively, correspond to M1-type and M2a-type macrophages. Meanwhile, both anti-inflammatory and fibrolytic macrophages probably belong to M2c-type macrophages [42, 59].

### 1.3. Roles of Macrophages in Anastomotic Leakage

As we previously discussed in our review, occurrence of AL mainly contains three factors: communication, infection, and healing disturbances. Communication means defect of the alimentary tract in the anastomotic region that connects the gastrointestinal lumina and abdominal cavity. Infection indicates anastomotic site bacterial infection. Healing disturbances include all substances that disturb a normal healing process such as hypoxia or inflammation. These three factors actively interact with each other: one factor takes place, and a responsive chain that consists of all factors will be initiated, eventually leading to AL. For example, infection provokes inflammatory response at the anastomotic site, which impairs collagen deposition [71, 72], then interferes with the normal healing process, and leads to a communication between the intra- and extraluminal gastrointestinal walls. On the contrary, communication allows the bowel content (including bacteria) to dislocate into the abdominal cavity, causes intra-abdominal infection, and afterwards delays anastomosis healing. Clinically, communication in some extent is regarded as a macroscopic clinical outcome, while infection and healing disturbances are durative biological processes. For AL, macrophages are mainly involved in the latter two mechanisms, which is also observed in other poorly healed wounds [73–77].

Anastomotic infection may be caused by anastomotic dehiscence (intestinal contents leak to the sterile abdominal cavity) or pre-/intraoperative contamination. Regardless of the cause of infection, in the contaminative infective environment, macrophages polarize into the M1 type as mentioned above. However, instead of supporting resistance to intracellular bacteria and controlling the acute phase of infection, an excessive or prolonged M1 program is deleterious for patients, as demonstrated in acute infections with *Escherichia coli* [78]. *E. coli* as a resident flora of the gut can induce a typical M1 profile through the recognition of lipopolysaccharides (LPS) by TLR4 [79, 80]. Classical activated M1-type macrophages upregulate the expression of inducible nitric oxide synthase (iNOS), which is responsible for the generation of nitric oxide (NO). NO was first identified to mediate arterial vasodilatation [81–83] and then was found to have a role in host defense against pathogens [84, 85]. Moreover, a prominent role has been described for NO in collagen deposition, fibrosis, and scar formation [71, 72, 86, 87]. High levels of wound NO, as in infection or inflammation, severely impair wound collagen synthesis [88]. Decreased deposition of collagen seriously weakens the anastomotic strength, which may lead to the failure of anastomotic healing. Therefore, improper M1 polarization of macrophages in bacterial infection of the abdominal cavity contributes to the occurrence of AL.

The role of macrophages in leakage with healing disturbances is more complicated. During a normal condition, tissue repair initiates from clearance of tissue debris and dead cells, efficiently phagocytosing those “tissue rubbish” by macrophages, and is critical for timely resolution of inflammation and successful healing. Nevertheless, for those patients complicated with diabetes mellitus, advancing in years, or undergone chemotherapy, the ability of macrophages to phagocytose is severely influenced, which directly leads to an accumulation of apoptotic or necrotic cells at the anastomosis site. This accumulation of dead cells prolongs the inflammatory phase, disturbs the healing process, and compromises the resolution of inflammation [73, 74]. Other disturbances such as ischemia or anastomotic hypoxia severely compromise the anastomotic healing [89, 90]. At a cellular level, exposing macrophages to an anoxic environment leads to the expression of proinflammatory cytokines like IL-1*β* and TNF-*α* and cytotoxic mediators like NO [91–93], which indicates that hypoxia can promote macrophages to polarize into the M1 phenotype. Excessively activated M1 macrophages sustain proinflammatory response and obstruct subsequent steps of the repair process that influences proper healing and remodeling of anastomosis [94–96], and the relevant mechanism is described above.

**Figure 2:**
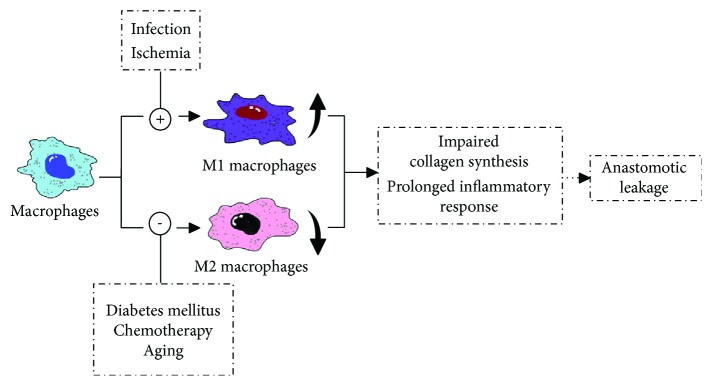
Imbalance of polarized macrophages contributes to the occurrence of anastomotic leakage. In some pathological conditions (infection, ischemia, diabetes mellitus, etc.), macrophages are abnormally activated into M1-type macrophages or inhibited to express the M2-type phenotype, which leads to long-time inflammatory response in the anastomotic site and influences collagen deposition and tissue repair; all of those are thought to be associated with anastomotic leakage.

Based on the available evidence, it seems that classical activated macrophages which express the M1 phenotype are responsible for the pathological process of defective anastomotic healing, whereas alternative activated macrophages which express the M2 phenotype play a critical role in inflammation resolution and successful tissue repair (Figure 2). Although M1-type macrophages participate in the early phase of normal wound healing, the programmed transformation of their polarized orientation from M1 to M2 lays the foundation of transient inflammatory response and the following tissue regeneration.

## 2. Conclusion

Macrophages are the most versatile immune cells and possess significant plasticity and heterogeneity. Macrophages can polarize into two main extremes and express corresponding phenotypes (M1 and M2). As polarization is the premise for macrophages to exert their diverse biological functions, different polarized macrophages play different roles in the physiological process of anastomotic healing and pathogenesis of AL. Reacquainting AL in the perspective of macrophages contributes to the exploration of new diagnostic tools and therapeutic targets. For example, in different recovery phases after anastomosis construction, the spectrum of cytokines and inflammatory mediators such as IL-1*β*, IL-6, IL-10, IL-12, TNF-*α*, ROS, and NO, which are secreted by macrophages, may appear an alteration. Moreover, the level of these substances could indirectly reflect the situation of an anastomosis. An abnormal fluctuation of these substances probably indicates disorder and defection of anastomosis healing, which can be regarded as premonition of AL. Because M1-type macrophages show a stimulating effect on AL and M2 macrophages are essential for anastomosis healing, regulation of M1/M2 polarization may find its therapeutic roles in the treatment of AL in the future.
